# Impact of Silica Ions and Nano Silica on Growth and Productivity of Pea Plants under Salinity Stress

**DOI:** 10.3390/plants11040494

**Published:** 2022-02-11

**Authors:** Lamiaa M. Ismail, Magda I. Soliman, Mohammed H. Abd El-Aziz, Heba M. M. Abdel-Aziz

**Affiliations:** 1 Botany Department, Faculty of Science, Mansoura University, Mansoura 35516, Egypt; lamyaaesmail@mans.edu.eg (L.M.I.); magdaisoliman@yahoo.com (M.I.S.); 2 Department of Genetics, Faculty of Agriculture, Mansoura University, Mansoura 35516, Egypt; mohahassan@mans.edu.eg

**Keywords:** antioxidants, gene expression, growth, *Pisum sativum*, silicon, nano-silicon

## Abstract

The present study was conducted to evaluate the effects of silicon (Si) and nano-silicon (NSi) on growth, yield, ions content, and antioxidant defense systems, including transcript levels of enzyme-encoding genes in *Pisum sativum* plants grown under salinity stress. Both Si and NSi were applied at the 3 mM level and NaCl was applied at 4 concentrations (100, 150, 200 and 250 mM). Vegetative growth, including plant height, leaf area, fresh and dry weights, and yield attributes were determined. Gene expression of antioxidant enzymes was analyzed, and their activities were determined. The results showed that salinity had deleterious effects on plant growth and yield. Salt-stressed plant leaves exhibited a greater activity of superoxide dismutase (SOD), peroxidase (POD), but a lower activity of catalase (CAT) when compared to the control. Na^+^ ions accumulated in roots and shoots of salinized plants. The application of Si and NSi significantly enhanced vegetative growth and relative water content (RWC), and caused significant increases in plant height, fresh and dry weight, total yield, and antioxidant defense systems. Si and NSi enhanced K^+^ content in roots and shoots under salinity treatment and decreased Na^+^ content in the studied tissues. It was concluded that the application of NSi was beneficial in improving the salt tolerance of *Pisum sativum* plants more than Si alone.

## 1. Introduction

Crop production losses are being caused by increased soil salinization, which is a major global problem [1]. Over 20% of the world’s arable land is affected, which reduces agricultural production [2]. Egypt has a persistent salinity issue as a result of its semi-arid zone weather, sea water, freshwater constraint, and climate change [3]. A plant’s production decreases dramatically when exposed to high levels of salinity, which affects nearly every aspect of its physiology and biochemistry. In Egypt and many other nations across the world, pea (*Pisum sativum* L.) is a significant and popular legume crop. With seeds containing 20–25% protein, 40–50% starch, and 10–20% fiber, it is high in protein, low in starch, and high in fiber [4,5]. It is consumed as immature seeds in Europe, Australia, America, and the Mediterranean, but as entire pods in Asia [6]. This plant is utilized in rotations with cereals for the supply of soil nitrogen, and is also used as a fodder crop for animals [7,8].

Even though salt stress reduces the yield of pea plants by 50% when NaCl is present at a concentration of 100 mM, pea yields were still lowered when the salinity was raised [9]. It is among the most oppressive constraints on agricultural yield due to soil and irrigation water containing excessive amounts of salt [10]. Osmotic stress, specific ion toxicity, imbalanced nutrition (in particular to accelerated uptake of Na^+^ over K ^+^), and oxidative stress are all believed to be factors contributing to saline-induced plant growth and productivity loss [11].

Stress due to salinity can have an impact on all stages of plant development, including seed germination and growth. Stress-induced adverse plant responses appear to be dependent on the medium’s osmotic and toxic effects as well as the stress’s intensity and duration [12]. In the case of salt stress, other mechanisms are known, such as the expulsion of excess salt as well as the translocation of salt and the production of osmoprotectants, whose purpose is to retain water inside the cells and protect proteins. There is evidence that nitrogen-containing compounds and osmoprotectants accumulate in stressed plants. The amount of nitrogen-containing compound accumulation varies by plant type and species [13]. Due to the impacts of salt stress on plants, along with the formation of reactive oxygen species (ROS), such as free radicals, hydroxyl radicals (.OH) and singlet oxygen (^1^O_2_), plants produce ROS such as superoxide anions (O_2_^•−^), hydroxyl radicals (.OH), and non-radical molecules like hydrogen peroxide (H_2_O_2_) [14]. Salt concentration and oxidative stress cause damage to a wide range of cellular components, including proteins, lipids, and DNA, thus the plant’s critical activities are disrupted [15].

Plants enhance their tolerance to unfavorable circumstances by simultaneously activating several defensive systems. Stress activates a huge number of genes, and many proteins are generated that further boost stress resistance [16]. Plants may combat oxidative damage by employing an effective antioxidant defense system that will sweep up ROS and safeguard the plants [14]. The fundamental phase of cellular defense is the antioxidant enzyme system, which comprises SOD as the leading component. It forms H_2_O_2_ and O_2_ from the extremely harmful superoxide ions (O_2_^•−^) by dismutating the O_2_^•−^ to H_2_O_2_. The first phase of ROS detoxification, which consists of using components of the ascorbate–glutathione cycle, is subsequently followed by a second step that uses the CAT, POD, and ascorbate peroxidase (APX) pathway, which includes the removal of H_2_O_2_ to form water and oxygen [13]. Glycine betaine, Mannitol, proline, and SOD, APX, CAT, and glutathione reductase (GR) have all been found to be overproduced in response to salinity stress [13].

One of the most important nutritional components for plant growth is silicon (Si) [17]. The potential of Si to improve plant development under salt stress is widely known [18]. Salinity resistance in many plant species, including wheat [19], barley [20,21,22], rice [23], cucumber [24], tomato [25], spinach [26], canola [27], maize [28], and *Cucurbita pepo* [29] has been reportedly mitigated by silicon. Several mechanisms of Si amelioration of salt stress have been proposed: (1) the accumulation of Na in the shoots of rice [23] and barley [20,21,22] after Si addition under salinity stress. (2) Reduction of Na uptake by the roots [30,31]. 

Because nanoparticles (NPs) have distinct physical–chemical characteristics in comparison to bulk particles, researchers are drawn to it [32]. As a result, nanomaterials demonstrate unique properties because of their minuscule size. Because of their increased surface area, the solubility and surface reactivity of their particles tend to be higher [33]. One of the practical nanomaterials shown to have a favorable influence in current agriculture is Nano-silicon (NSi) [34]. In contemporary agriculture, NSi has been found to be advantageous [34,35,36]. However, there is still a lack of understanding of how NPs affect plant growth and development [35]. With the use of NSi, Larix seedlings developed and increased in quality [37] while drought-stressed strawberry plants increased in productivity [36]. Salinity and silicon in higher plants have been studied extensively, but little is known about the potential advantages of NSi to reduce and alleviate salt stress damage in plants [38].

Therefore, the current study aimed to elucidate the potential effect of the application of Si (3mM) and NSi (3mM) on growth, development, productivity, ion selectivity, and the expression of antioxidant genes and antioxidant enzymes activities of pea (*Pisum Sativum* L.) under salinity stress conditions compared to untreated plants. The hypothesis was to try to confirm whether Si or NSi is more effective in alleviating salinity stress in pea plant. 

## 2. Results

### 2.1. Plant Vegetative Growth

In comparison to the control, treatment of pea seedlings with Si induced slight increase in shoot and root length, shoot and root fresh weight, shoot and root dry weight, number of leaves, and leaf area at the vegetative stage whereas more beneficial effect was recorded after NSi application. Under salt stress, shoot length, shoot and root fresh weight, shoot and root dry weight, and leaf area were gradually decreased by increasing salt concentration when compared with control, while root length was increased compared to that of the control. The lowest values of growth parameters were recorded at the highest level of salt concentration (150, 200 and 250 mM). At low salt stress (100 mM), no significant effect on fresh and dry weights was observed. However, the application of Si and NSi alleviated salinity –inducing a reduction in growth and significantly increasing shoot and root length, shoot and root fresh weight, shoot and root dry weight, the number of leaves, and leaf area when compared with the corresponding level of salinity (Table 1). 

### 2.2. Yield Components

Data registered in Table 2 revealed that increasing salinity level resulted in a gradual decrease in pod length, pod weight, number of pods, and crop yield per plant. Similarly, salinity stress significantly reduced number of seeds per pod, seed weight per pod, total seed yield, and straw yield; the reduction was much greater at salinity levels of 200 and 250 mM than at 100 or 150 mM. In this respect, 250 mM NaCl treatment caused a reduction of about 24.51%, 68.84%, 66.67% and 64.48% in pod length, pod weight, number of pods, and crop yield, as compared with the control, respectively. Reductions in the number of seeds per pod, seeds weight per pod, total seed yield and straw yield at 250 mM were severe and reached about 28.57%,86.12%, 85.46% and 60.65% as compared to the control, respectively. 

On the other hand, Si and NSi applications significantly overcame the adverse effects of salt stress and increased all yield components in saline treated or untreated plants (Table 3). In this regard, Si treatment caused an increase in pod weight, crop yield per plant, and straw yield by about 31.97%, 13.60% and 2.50%, respectively, as compared to the control (Table 3). The corresponding increases with NSi treatment were about 61.66%, 44.46% and 26.58%, respectively. Concerning seeds weight per pod and total seed yield per plant, the Si treatment caused an increase of about 12.46% in seeds weight per pod and about 21.66% in total seed yield per plant, while NSi treatment caused an increase of about 11.05% and 17.21% in seeds weight per pod, and total seed yield per plant, respectively. 

The harvest index (HI) and crop index (CI) were affected significantly by salinity stress (Table 3). In this regard HI and CI values were decreased with increasing salinity levels. At 250 mM of NaCl, the harvest and crop indices of silicon untreated plants were decreased by about 58.33% and 50.00%, respectively, as compared to 0 mM NaCl treatment. It is clear from the data in the same table that Si and NSi treatments had a significant enhancing effect on seed yield of pea; therefore, the harvest index values were increased with Si and NSi treatments under different salt treatments. 

### 2.3. Ion Selectivity

Examination of Table 4 revealed that Na^+^ content increased significantly in both roots and shoots of plants under salinity treatments while it decreased significantly under Si and NSi treatments. The magnitude of response was more pronounced with the NSi treatment. On the other hand, K^+^ content decreased significantly under salinity conditions in both roots and shoots but Si and NSi alleviated this effect under salinity conditions with NSi being superior over Si. In a similar manner Na^+^/K^+^ ratio showed increased values under salinity treatments alone, and relatively lower values with Si and NSi treatments (Table 4).

### 2.4. Expression of Antioxidant Defense Genes

**Figure 1:**
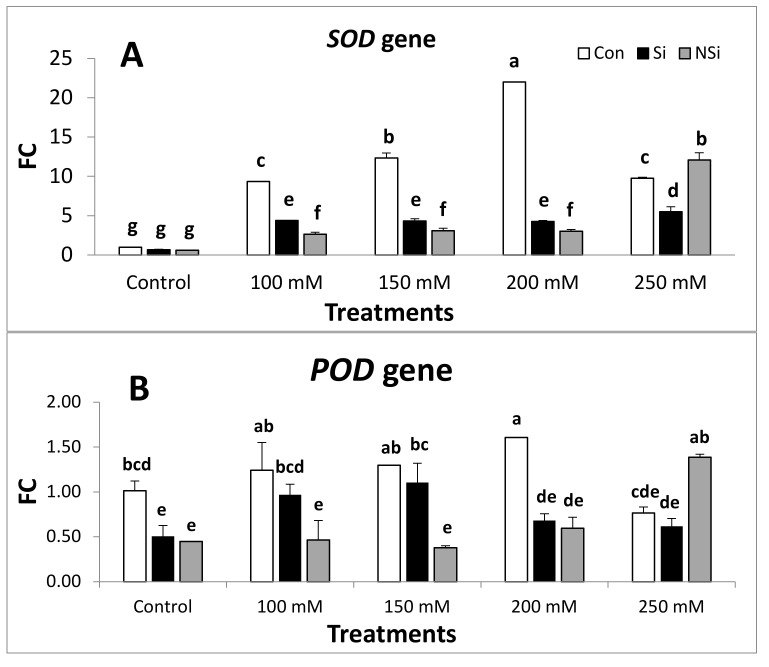

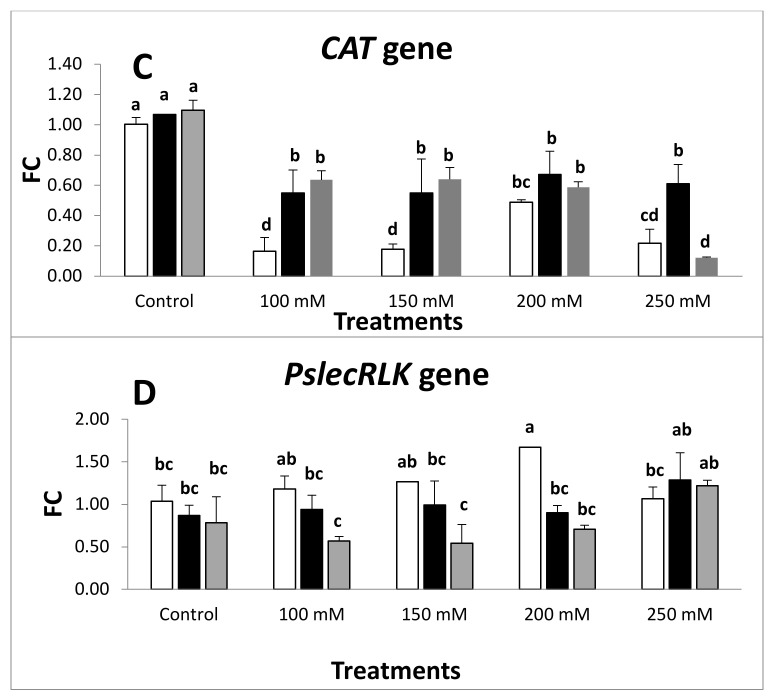
Relative expression pattern of (**A**) SOD gene, (**B**) POD gene, (**C**) CAT gene and (**D**) PslecRLK gene in salt-stressed pea seedlings in the presence or absence of Si or NSi. Vertical bars represent the standard error (±S.E.). Means denoted by similar letters are not significantly different at the 5% probability level-using Post Hoc Duncan test.

The expression patterns of four genes: SOD, POD, CAT and PslecRLK in the leaf tissue were analyzed using qRT-PCR and the results are represented in Figure 1. Two reference genes (RGs), Act and Tub, were included to significantly stabilize gene expression level calculations from the qRT-PCR data, and the Pearson correlation analysis was made by SPSS software. The relationship between the C_T_-values of these two RGs was a linear and highly significant positive correlation (r = 0.836 **). Therefore, to further enhance validity and reproducibility of relative expression quantifications, the C_T_ of a single RG in DCT was replaced with an averaged C_T_ value from the two RG. The transcript level of the target genes was normalized by the average C_T_ value of the two reference genes. Antioxidant defense genes showed differential expression patterns in mRNA expression under salt stress either in the presence or absence of Si and NSi. 

Analysis of the SOD gene indicated that the relative transcript levels of this gene (Figure 1) was significantly upregulated in salt-stressed seedlings at all concentrations when compared with the non-stressed control seedlings. However, the application of either Si or NSi to pea seedlings under salt stress induced a significant downregulation in the expression of SOD gene when compared with the corresponding level of salinity except at 250 mM level of salinity, the application of NSi showed a significant upregulation in the expression of SOD gene when compared with 250 mM salinity treatment. Pea seedlings treated with either Si or NSi alone showed a non-significant difference in the transcript level of SOD gene when compared with control. 

The analysis of POD (Figure 1) gene exhibited the same expression pattern of SOD gene and showed that the relative transcript levels of this gene was significantly upregulated in salt-stressed seedlings at all concentrations except for 250 mM was non-significantly decreased when compared with the non-stressed control seedlings. However, the application of either Si or NSi to pea seedlings under salt stress induced a significant downregulation in the expression of the POD gene when compared with the corresponding level of salinity except at 250 mM level of salinity, The application of NSi showed a significant upregulation in the expression of the POD gene when compared with the 250 mM salinity treatment. Pea seedlings treated with either Si or NSi alone showed a significant decline in the transcript level of the POD gene when compared with the control. 

In contrast, an examination of the CAT gene revealed a different expression pattern (Figure 1) and showed that the expression level of this gene was significantly downregulated in salt-stressed seedlings at all concentrations when compared with the non-stressed control seedlings. However, the application of either Si or NSi to pea seedlings under salt stress induced a significant upregulation in the expression of CAT gene when compared with the corresponding level of salinity except at 250 mM level of salinity; the application of NSi showed a non-significant downregulation in the expression of CAT gene when compared with 250 mM salinity treatment. Pea seedlings treated with either Si or NSi alone showed a non-significant rise in the transcript level of the CAT gene when compared with the control. 

The analysis of PslecRLK gene (Figure 1) indicated the same pattern of expression of the SOD and POD genes and indicated that the relative transcript levels of this gene was significantly upregulated in salt-stressed seedlings at all concentrations when compared with the non-treated control seedlings. However, the application of either Si or NSi to pea seedlings under salt stress induced a significant downregulation in the expression of PslecRLK gene when compared with the corresponding level of salinity except at 250 mM level of salinity; the application of Si and NSi showed a non-significant upregulation in the expression of PslecRLK gene when compared with 250 mM salinity treatment. Pea seedlings treated with either Si or NSi alone showed a non-significant decrease in the transcript level of PslecRLK gene when compared with the control.

### 2.5. Antioxidant Enzyme Activities

**Figure 2:**
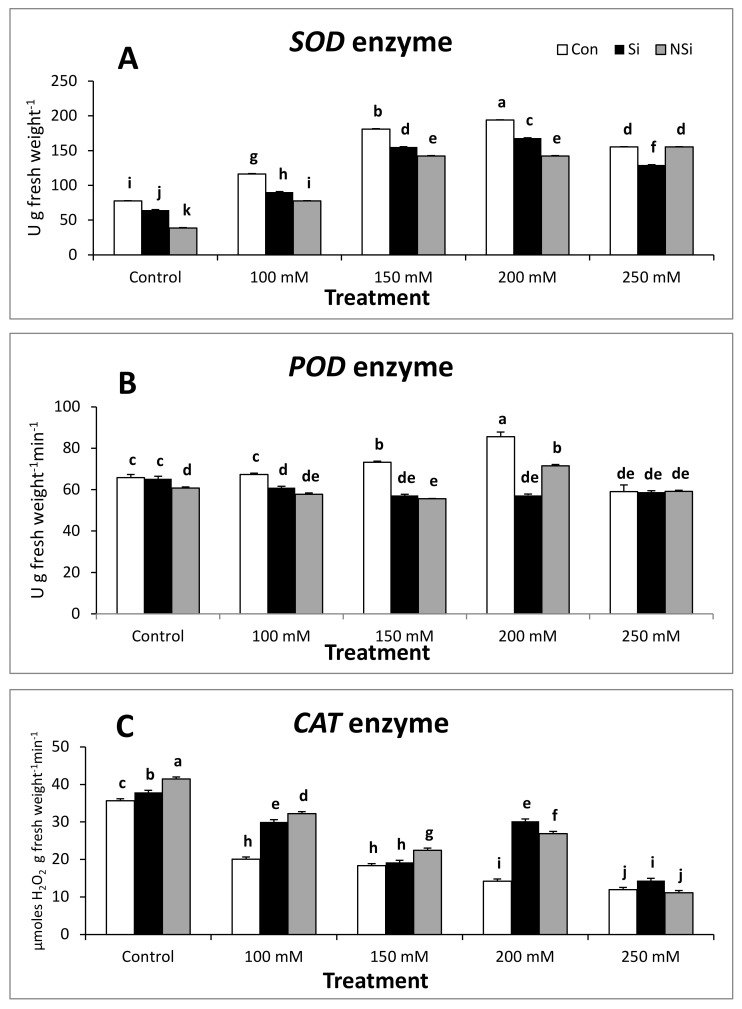
The activity of (**A**) SOD, (**B**) POD and (**C**) CAT enzymes in salt-stressed pea seedlings in the presence or absence of Si or NSi. Vertical bars represent standard error (±S.E.). Means denoted by similar letters are not significantly different at the 5% probability level-using Post Hoc. Duncan test.

The activity of SOD and POD enzymes was significantly enhanced in salt-stressed seedlings at all concentrations relative to control seedlings. However, the application of either Si or NSi to pea seedlings under salt stress induced a significant decline in the activity of SOD and POD enzymes when compared with the corresponding level of salinity. Pea seedlings treated with either Si or NSi alone showed a significant decrease in the activity of SOD and POD enzymes when compared with the control (Figure 2). 

In contrast, CAT activity was significantly decreased in salt-stressed seedlings at all concentrations relative to control seedlings. However, the application of either Si or NSi to pea seedlings under salt stress ameliorated the inhibitory effect of NaCl by enhancing the activity of CAT enzyme compared with the corresponding level of salinity. Pea seedlings treated with either Si or NSi alone showed a significant increase in the activity of the CAT enzyme when compared with the control (Figure 2). 

## 3. Discussion

Salinity stress is one of the most important environmental variables restricting the productivity of agricultural crops [39]. The results of the present study revealed that salinity had a negative effect on growth parameters measured in terms of shoot length, shoot and root fresh weight, shoot and root dry weight, and leaf area. It is also clear that relative water content (RWC) was decreased significantly with NaCl treatments. These results agree with those reported by [40,41].

The recorded suppression of plant growth under saline conditions was attributed to limited availability of water and/or to toxicity of sodium chloride [39]. Reduced dry weight under salinity stress was ascribed to inhibition of food formation and translocation, inhibition of certain metabolic processes, inhibition of chloroplast formation, production of ethylene and abscisic acid, and decreasing nutrient absorption [42]. Growth reduction due to salt-induced osmotic effect has been reported in a number of plants, such as soybean [43], pepper [44], wheat [45], and pea [46].

Although silicon (Si) is regarded as a non-essential element [47], results from some earlier studies show that the exogenous application of Si can stimulate growth of most plant species [48]. This increase in plant growth as a result of Si application not only takes place under normal growth conditions [49,50], but also under stressful conditions [51,52,53]. This effect of Si on plant growth is dose and crop specific [54]. Silicon affects plant growth under stressed conditions by affecting a number of processes, including an improvement in plant water status [55], changes in the ultra-structure of leaf organelles [56], upregulation of plant defense systems [26], and mitigation of the specific ion effect of salt [57].

In the present study, application of Si significantly increased the growth and morphological attributes of pea seedlings under salt treatments. This silicon-induced improvement in plant growth under salt stress may be due to the significant role of Si in the improvement of plant water status [55]. Such beneficial effects of Si on plant biomass production under salt stress as well as non-saline conditions were also detailed in some other plant species such as wheat (*Triticum aestivum*) [57], mesquite (*Prosopis juliflora*) [58], and zinnia (*Zinnia elegans*) [59], indicating the beneficial effects of Si application in alleviating salt-induced inhibitory consequences on plant growth.

It is important to consider the role of Si in plant water status when analyzing the beneficial effect of Si under saline growth conditions, because the initial decline in plant growth after salt imposition is due to the osmotic effect of salts [60]. The ability of a plant to retain water under saline growth conditions can improve tissue tolerance mitigating an excessive ion concentration by a dilution effect [61]. It was obvious that water content of NaCl treated plants was increased with Si and NSi treatments, but the increase in shoot and root water content under NSi treatments was more observed than that recorded with Si treatments. In this regard, it was reported [62] that the application of Si improved water economy and dry matter yield of wheat plants. Some evidence shows that silicon may be involved in the osmotic adjustment of numerous plant species [63]. In this regard, it was demonstrated that in *Vicia faba* the treatment of 75 and 100 mM NaCl plus 1 mM silicon increased biomass to 14% and 18% compared with the salt treatment without silicon [64].

Among others, one important factor inducing the deleterious effects of salt is the physiological drought generated by the high NaCl concentration in the apoplast [65,66]. It is known that silicon is actively absorbed through the roots in the form of uncharged monosilicic acid, and is then passively transported by the transpiration stream, and irreversibly precipitated as SiO_2_-nH_2_O in cells walls and cell lumens of all plant tissues [67,68]. Thus, the ameliorative effect of silicon could be related to the hydrophilic nature of silicone. SiO_2_-nH_2_O deposition could help to keep water, to dilute salts and to protect tissues from physiological drought. Thus, most likely, silicate crystal deposition beneath the epidermal cells of leaves and stems could have reduced water loss in pea as has been shown in other crops such as rice, sugarcane, turfgrass, or tomato. The higher plant water content of salinized plants treated with Si could have contributed to salt dilution and consequently to reduce deleterious effects of saline ions thus explains the increase in plant growth [68]. 

In the present study, the vegetative growth and morphological attributes of salt-treated pea plants were increased with Si and NSi treatments but the increase under NSi treatments was more observed than that recorded with Si treatments. Particle size is of great importance in particle adhesion and interaction with biological cells [69] as well as in the pathway of cellular uptake [70]. Furthermore, a strong relationship was found between the extent of particle uptake and particle size. However, the role of nanoparticles and their modes of action in plant growth and development are not yet well known. Their high surface to volume ratio increases their reactivity, and it is possible that the biochemical activity of nanoparticles might play a role in their action [71]. Nano silica is more easily absorbed by plants than inorganic silica, thus justifying the greater protective effect of SiNp than Si under salt stress in pea seedlings [72].

Yield components of pea such as pod length, pod weight, number of pods per plant, number of seeds per pod, and total seed yield per plant all play an important role in determining the final crop yield. The reduction in yield attributes noted in the current study under NaCl stress may be attributed to the inhibitory effects of salinity on some metabolic processes in plant tissues [42]. It was shown that at the high salinity level, salts may build up in the apoplast and dehydrate the cell and suppress enzymes involved in carbohydrate metabolism in cytoplasm or may exert a direct toxic effect in chloroplast and photosynthetic processes [39]. It has already been reported that salt stress causes a reduction in the yield of various crops [73,74]. The reduction in total seed weight under saline stress may be ascribed to the inhibition in uptake and transfer of nutrition materials during the growth of grains and their filling periods. Moreover, salinity may cause severe damage to the ovary, resulting in fruit drop and a reduction in yield [39].

On the other hand, Si and NSi applications significantly overcame the adverse effects of salinity stress and increased all yield components in both saline treated or untreated plants (Table 3). In this respect, silicon was reported to enhance growth and yield of many of higher plants particularly under biotic and abiotic stresses [57,75]. A number of possible mechanisms are proposed by which Si can enhance the resistance of plants against salt stress, which is the major yield limiting factor in arid and semiarid areas [75]. The increase in seed yield after silicon amendment in mild saline conditions may indicate an improvement in the translocation of minerals and metabolites necessary for seed setting. The role of silicon as a beneficial mineral nutrient for reproductive growth of plant is well documented [76]. Generally, Increased K^+^ uptake and decreased Na^+^ uptake by Si treatments was reported to be the major mechanisms responsible for better growth and yield of plants under salinity [27].

The harvest index (HI) and crop index (CI) were decreased with increasing salinity levels. However, the harvest index values were increased with Si and NSi treatments under different salt treatments. In this regard, it was stated that abiotic stress decreased the harvest index [77]. Silicon treatment, on the other hand, improved the harvest index of different crops under salinity stress [78]. Furtheremore, it was found that under salt stress conditions, Si treatments increased yield, yield components, and the harvest index of pea crops [75,79]. The present study displayed that NSi treatments were the most effective in reducing the negative effect of salinity on the harvest index. In another study [80], researchers found that water deficit stresses decreased the harvest index while titanium dioxide nanoparticles caused the maximum harvest index, whereas titanium oxide (bulk) and control treatments were similar.

The generation of reactive oxygen species (ROS) by plants is a common phenomenon under stress conditions. These stress-generated ROS damage macromolecules, such as DNA, proteins, and lipid structures [81]. To scavenge ROS, plants have an internal protective enzyme catalyzed clean-up system, thus ensuring normal cellular function [16]. Thus, the observed increase in SOD and POD activities in pea plants under salinity stress may be due to the endogenous defense mechanisms activated by plants to mitigate oxidative stress-associated damage [82]. The results of the present study are in accordance with the findings of [83] who found that SOD and POD enzyme activities were increased in *Pisum sativum* seedlings grown from NaCl-treated (1 or 10 mM) seeds. Similarly, it was found that the activities of antioxidant enzymes superoxide dismutase (SOD), ascorbate peroxidase (APX), and catalase (CAT) were up-regulated in pea plants under salinity stress [16]. Likewise, [84] analyzed the antioxidant enzyme activity in pea and found a prominent increase in SOD, POD and CAT under saline mediums. 

However, the application of either Si or NSi to pea seedlings under salt stress induced a significant decline in either the relative transcript level or the activity of SOD and POD enzymes when compared with the corresponding level of salinity. Si application in crops during abiotic stress conditions can regulate ROS generation [85]. It was shown that silicon (Si) application to rice root zones influenced the hormonal and antioxidant responses under salinity stress. The results showed that Si treatments significantly increased rice plant growth compared to controls under salinity stress. Si treatments reduced the sodium accumulation resulting in low electrolytic leakage and lipid peroxidation compared to control plants under salinity stress [86]. Enzymatic antioxidant (catalase, peroxidase, and polyphenol oxidase) responses were more pronounced in control plants than in Si-treated plants under salinity stress [86]. It was observed that the application of Si in rice plants under salinity stress significantly decreased the activities of non-enzymatic MDA and enzymatic antioxidants POD, PPO, and CAT [87]. On the other hand, it was observed that when they applied Si to borage plants, SOD activity was significantly increased but activity of CAT and APX was slightly decreased [88].

In soil, Silicon (Si) is the second most abundant element. Even though Si is present in soil in different forms, plants can easily absorb silicic acid Si(OH)_4_ from soil. After being absorbed from the soil into the root, it gets translocated to the shoot area via the xylem, where it can stimulate various physiological responses, such as plant growth and development [48,89], enzymatic activity [48,90], and gene expression [91]. After uptake, Si accumulates on the epidermis of many tissues primarily as a polymer of hydrated amorphous silica [53]. In leaves, silicic acid is converted into insoluble silica which accumulates just beneath the cuticle [92], forming a Si-cuticle double layer. It has been suggested that Si deposition in the exodermis and endodermis may reduce sodium uptake under salinity by reducing apoplastic transport across the root [25,93]. Silica deposition in the leaf reduces transpiration and any decreases in transpiration could reduce sodium uptake [25].

Most of the beneficial effects of Si in mitigating abiotic stresses, such as salt stress, are attributed to Si deposition in the cell walls of roots, leaves, stems and hulls [94]. For example, deposition of Si in the roots reduces apoplastic bypass flow and provides binding sites for metals, resulting in decreased uptake and translocation of toxic metals and salts from the roots to the shoots [94]. Saline ions are continuously transported to the aboveground plant by transpirational flow, inducing deleterious effects in the tissues when the saline ion content reaches a toxic threshold. Any factor that can delay attainment of the toxic threshold is important for increasing tolerance to salinity [94]. It was reported that silica deposition in the leaf limits transpiration in *Prosopis juliflora* and wheat, and thus, salt accumulation [21,58]. It was suggested that silicate crystals deposited in epidermal cells form a barrier that reduces water loss through the cuticles [95]. The ability of a plant to retain water under saline growth conditions can improve tissue tolerance, alleviating an excessive ion concentration by a dilution effect [61]. The higher plant water content of salinized plants treated with Si could have contributed to salt dilution, and consequently reduced the deleterious effects of saline ions.

Thus, the decrease in in the activity of SOD and POD enzymes observed in the present study following Si application could be related to salt dilution as a consequence of the higher water content we found in salinized pea plants treated with Si, which may result from Si deposition in the cell walls and endodermis, thereby reducing Na^+^ uptake through a reduction in apoplastic transport across the root and its translocation to the shoots [93]. 

In contrast, the relative transcript level and the activity of CAT were significantly decreased in salt-stressed seedlings relative to the control. Similar results were reported in soybeans [96]. The decline in CAT activity under salinity has been reported by several researchers [97], which ultimately leads to oxidative stress [98,99]. The decline of CAT activity under stressful condition may be due to a reduced rate of protein turnover [97]. It could also be attributed to enhancement of H_2_O_2_ levels [100]. Reduced growth of the salt-treated pea plants might have resulted from alterations to the activity of this ROS-scavenging enzyme. However, the application of either Si or NSi to pea seedlings under salt stress induced a significant rise in the activity of CAT enzyme. Higher catalase activity under salinity following Si application has already been observed in tomato, barley, and canola plants [27,29,90,101]. One of the adaptive traits under salinity stress is an increase in catalase (CAT) activity which reduces the toxic levels of H_2_O_2_ and protects the cell from oxidative damage [102]. It was concluded that Si supplemented plants showed resistance to abiotic stress through lowering ROS production by enhancing CAT and APX activities, as both are involved in the conversion of H_2_O_2_ into H_2_O [103].

## 4. Materials and Methods

### 4.1. Plant Material and Growth Conditions

Seeds of pure strain of pea (*Pisum sativum* L. cv. Intisar 1) were kindly provided by the Central Administration for Seed Production, Horticulture Research Institute, Agriculture Research Center, Ministry of Agriculture, Giza, Egypt. Silicon used was in the form of sodium silicate (commercially available). Powdered SiO_2_ nanoparticles were purchased from NanoTech Egypt for Photo-Electronics (size 5–9 nm). A pot experiment was conducted in three seasons in the Botanic Garden of the Faculty of Agriculture, Mansoura University in the periods from November 2018 to February 2019, November 2019 to February 2020 and November 2020 to February 2021 under natural environmental conditions (mean temperature 24 ± 2 °C and relative humidity 60%). 

Healthy and proximate equal-sized seeds of pea (*Pisum sativum* L. cv. Intisar 1) were selected, pretreated with 0.001M mercuric chloride (HgCl_2_) solution for 1 min for seed surface sterilization, and then washed thoroughly with tap water and soaked for 3 h in tap water. Seeds were sown in clean plastic pots (22 cm in diameter) containing clay-sandy soil (2:1 *v/v*). All pots contained equal amounts (7 kg) of homogenous soil in which 10 seeds were planted. Pots were divided into three groups. The first group was left as control. The second group was for Si treatment. The third group was for NSi treatment. Silicon and Silica (Silicon Dioxide) Nanoparticles were soil applied both at a concentration of 3 mM (This concentration was determined to be optimum for growth of pea plants in preliminary expermints). After two weeks from sowing and when the seedlings reached the second leaf stage of growth, the soil in the first group was irrigated to field capacity (500 mL) with water while the soil in the second group was supplied with 500 mL of sodium silicate solution whereas in the third group, the soil was added with 500 mL nano silica suspension. The treatments were applied for 2 weeks (every 5 day). After 5 days from the last treatment and at the fifth leaf stage, each group was divided into five subgroups according to salt irrigation as follows: 0, 100, 150, 200, and 250 mM NaCl solution. Thus, a total of fifteen treatments represented all planned possible applications of penta-replicated in a completely randomized design. Each treatment was represented by five replicates (each replicate was one pot containing six plants). Salt treatments were imposed to 30-day-old seedlings by adding 250 mL NaCl solution to the soil. The salt irrigation persisted for 2 weeks (5 day interval). The harvest of the vegetative stage was performed after 15 days of the salt treatment; plant samples were collected for estimation of growth parameters, estimation of ions, estimation of antioxidant activity and biochemical and gene expression analyses. 

The remaining seedlings were allowed to reach to flowering stage and yield stage. All pots were irrigated with tap water according to the usual practice and maintained at room temperature.

It should be mentioned that the results for growth and yield components and analysis of the wide array of metabolites either at vegetative stage, flowering stage or yield obtained in the three large-scale experiments were remarkably close, thus only the mean values obtained from the three large-scale experiments will be presented in the corresponding tables and figures given in this study. 

### 4.2. Estimation of Growth and Yield Parameters

Growth was assessed in terms of shoot and root length, shoot and root fresh weight, shoot and root dry weight, number of leaves/plant, and leaf area/plant. Seedlings were selected randomly from control and treated samples, divided into root and shoot and their lengths were measured using a meter scale. After recording the root and shoot fresh weights with the help of an electronic balance, the samples were oven-dried at 80 °C for 48 h to determine the dry weight. Leaf area was measured using image j.

Yield was measured in terms of shoot length, plant height, pod length, pod weight, crop yield/plant, number of pods/plant, number of seeds/pod, seeds weight/pod, total seed yield/plant, straw yield, harvest index, crop index and mobilization index:

Harvest index = economic yield (seed yield)/straw yield [104];

Crop index = seed yield/biological yield (seed yield+ straw yield) [104];

Mobilization index = crop index/straw yield [105]. 

### 4.3. Estimation of Ions

Ions contents were measured according to the method described in [106]. Oven-dried roots and shoots of pea plants (vegetative stage) were ground into a fine powder. For the wet digestion, 10 cm^3^ of conc. HNO_3_ were added to a known weight of each dry ground sample and the mixture was heated at 80–90 °C on a hotplate in a fuming hood. Heating was continued until the production of red NO_2_ fumes ceased. The contents were further heated until the volume was reduced to 3–4 cm^3^ and became colourless, but it should not be dried. After cooling the contents, the volume was made up with distilled water and filtered through Whatman No. 1 filter paper. This solution was used for nutrient estimation. Na and K contents were analyzed by a flame photometer (Jenway-PFP7, Essex, UK). 

### 4.4. Gene Expression Analysis

At the end of the vegetative stage, fresh leaf samples were collected, covered with aluminum foil, frozen immediately in liquid nitrogen, and stored at −80 °C until used for gene expression analysis using Quantitative Real-Time PCR (qRT-PCR). Total RNA was isolated from leaf samples using RNeasy Plant Mini Kit (Qiagen GmbH, Hilden, Germany). The concentration and purity of the RNA were assayed spectrophotometrically at 260 and 280 nm (Nanodrop Technologies, Wilmington, DE, USA). The total RNA was then evaluated for quality on an agarose gel. For RT-PCR, first-strand cDNA was reverse transcribed from 1 μg RNA with oligo (dT) primers, using a RevertAid First Strand cDNA Synthesis Kit (Fermentas GmbH, St. Leon-Rot, Germany) according to the manufacturer’s instructions. Based on previous studies, gene-specific primers were synthesized and used to amplify cDNA of target and reference genes of interest in RT-PCR reaction. Primer sequences of the salt stress-related genes (target genes) and two housekeeping genes (Actin and tubulin) as reference genes in pea plants are presented in Table 5. RT–PCR was carried out in triplicates on the iCycler Thermal Cycler (Bio-Rad Laboratories, Hercules, CA, USA) using the Maxima SYBR Green/ROX qPCR Master Mix (2X) manufacturer’s instructions. The conditions for PCR amplification were 95 °C for 10 min; 40 cycles of 95 °C for 15 s and 60 °C for 60 s. Melting curve analysis was applied to test the amplification specificity, this curve was conducted from 55 °C to 95 °C to confirm the amplification of a single amplicon. Cycle threshold (C_T_) values from housekeeping (Act and tub) pea genes were used for qRT-PCR data normalization and the relative expression levels were determined using 2^−∆∆Ct^ method [107].

### 4.5. Estimation of Antioxidant Enzyme Activities

#### 4.5.1. Enzyme Extraction

Crude enzyme extracts were prepared by grinding 0.2 g of 45-day-old fresh pea leaves from control and treated seedlings in 2 mL of chilled 0.02 M phosphate buffer (pH 7) using a pre-chilled mortar and pestle. The homogenate was transferred to falcon tubes and the volume was adjusted to 11 mL. The tubes were then centrifuged at 5000 rpm for 10 min at 4 °C. The resulting supernatant served as the enzyme extract for the determination of SOD, CAT, POD activities [108].

#### 4.5.2. Estimation of Superoxide Dismutase Activity (SOD, EC1.11.1.7)

In the present study, SOD activity was estimated according to the method of [109]. As applied by [78], one SOD activity enzyme unit is classified as the level of enzyme needed to inhibit nitro-blue-tetrazolium (NBT) reduction at 560 nm by 50%. To start the reaction, the reaction mixture comprised of: 1 cm^3^ of working buffer (50 mM phosphate buffer; pH 8.5), 1 cm^3^ of 1 mM NBT and 1 cm^3^ of 1 mM NADH, 0.1 cm^3^ of enzyme extract, blended well, and 0.1 cm^3^ of 0.1 mM of phenazine methosulphate. SOD activity was measured by considering the ability of the enzyme extract to inhibit NBT photochemical reduction.

#### 4.5.3. Estimation of Peroxidase Activity (POD, EC1.11.1.7)

Peroxidase catalyzes the oxidation of various hydrogen donors in the presences of H_2_O_2_, Peroxidase activity was assayed by the method of [110].1 mL of enzyme extract was added to 3 mL of pyrogallol in 0.1 M phosphate buffer and 0.5 mL of 1% H_2_O_2_. The mixture was shaken well and incubated for 1 min. the reaction was terminated by the addition of 1 mL of 2.5M H_2_SO_4_. The absorbance was read at 420 nm against the blank to determine the amount of formed purpurogallin. The enzyme activity was expressed in units. One unit was defined as unit per g fresh weight per min.

#### 4.5.4. Estimation of Catalase Activity (CAT, EC1.11.1.6)

Catalase (CAT) activity was assayed by the method of [111]. The enzyme extract (0.5 mL) was added to the reaction mixture containing 1 mL of 0.01 M phosphate buffer (pH 7.0), 0.5 mL of 0.2 M H_2_O_2_, 0.4 mL H_2_O and incubated for one minute. The reaction was terminated by the addition of 2 mL of acid reagent (5% potassium dichromate/glacial acetic acid mixture, 1:3 by volume). To the control, the enzyme was added after the addition of acid reagent. All the tubes were heated for 10 min and the absorbance was read at 610 nm. Catalase activity was expressed in μmoles of H_2_O_2_ consumed/min/g fresh tissue. 

### 4.6. Statistical Analysis

It should be mentioned that all the results obtained in the three experiments were remarkably close, thus only the mean values obtained from the three large-scale experiments will be presented in the corresponding tables and figures given in this study. All experimental data were subjected to analysis of variance (ANOVA) using CoStat software. ANOVA was conducted using one-way ANOVA in a fixed factor with full factorial analysis. For comparison between groups, Duncan’s test was applied at a 0.05 probability level.

## 5. Conclusions

It is evident that the use of nano-silicon enhanced pea crop productivity under salinity stress, with the magnitude of response being evident with 100 and 150 mM NaCl. Thus, we recommend the use of NSi to improve salinity tolerance of pea plants in moderately saline soils. Si appeared to nullify the harmful effects of salinity stress on pea plants at higher concentrations (200 and 250 mM NaCl). So, it is recommended to use Si in extreme saline soils to grow pea plants. Further studies are needed at the field level, and to investigate the toxicity of the used nano-silicon to animals and humans.

## Figures and Tables

**Table 1:** Effect of silicon and nano-silicon on growth of *Pisum sativum* plants (vegetative stage) exposed to salinity stress. Means (of three replicates), in each column, followed by similar letter are not significantly different at the 5% probability level-using Post Hoc. Duncan test.

Treatment	Shoot Length (cm plant^−1^)	Shoot Fresh Weight (g plant^−1^)	Shoot Dry Weight (g plant^−1^)	Shoot Water Content (g plant^−1^)	Root Length (cm plant^−1^)	Root Fresh Weight (g plant^−1^)	Root Dry Weight (g plant^−1^)	Root Water Content (g plant^−1^)	No of Leaves/Plant	Leaf Area (cm^2^ plant^−1^)
Control	26.60 bcd	4.76 bcd	0.89 cdefg	3.87 abcdef	3.45 ab	0.20 abcd	0.03 bc	0.17 ab	6.00 b	57.0 l
Si	28.53 ab	6.28 ab	1.07 bc	5.21 ab	4.67 ab	0.25 ab	0.03 bc	0.21 a	7.00 ab	70.62 e
NSi	30.40 a	7.01 a	1.68 a	5.33 a	4.83 ab	0.26 a	0.05 a	0.21 a	8.00 a	68.98 f
100 mM	22.25 fghi	4.37 cde	0.88 cdefg	3.49 bcdef	3.80 ab	0.14 cdef	0.03 cde	0.11 bcd	7.00 ab	55.04 m
100 mM + Si	26.20 bcde	5.78 abc	1.02 bcd	4.76 abc	3.50 ab	0.18 bcde	0.03 bc	0.14 abc	8.00 a	60.09 i
100 mM + NSi	27.30 bc	6.39 ab	1.30 b	5.09 abc	3.15 b	0.21 abc	0.04 ab	0.17 ab	8.00 a	61.12 h
150 mM	22.00 fghi	4.22 cde	0.68 defgh	3.54 bcdef	4.80 ab	0.12 def	0.03 cde	0.09 cd	7.00 ab	49.94 n
150 mM + Si	26.00 bcde	5.57 abc	0.91 cdef	4.66 abcd	4.00 ab	0.16 cdef	0.03 bc	0.12 bcd	8.00 a	88.65 a
150 mM + NSi	24.50 cdef	4.01 cde	0.62 efgh	3.39 cdef	3.97 ab	0.19 bcde	0.03 bcd	0.15 abc	8.00 a	78.89 b
200 mM	21.00 ghi	2.95 e	0.51 h	2.44 ef	5.00 ab	0.09 f	0.02 de	0.07 d	7.00 ab	36.95 o
200 mM + Si	23.27 efgh	3.55 de	0.57 fgh	2.98 def	4.25 ab	0.14 cdef	0.03 cde	0.11 bcd	8.00 a	73.88 d
200 mM + NSi	23.30 efgh	5.68 abc	0.95 cde	4.73 abc	4.53 ab	0.14 cdef	0.02 cde	0.11 bcd	7.00 ab	76.49 c
250 mM	19.50 i	2.74 e	0.53 gh	2.21 f	5.63 a	0.11 ef	0.02 e	0.09 cd	7.00 ab	59.18 k
250 mM + Si	20.50 hi	3.29 de	0.57 fgh	2.72 ef	4.90 ab	0.13 def	0.03 cde	0.10 bcd	8.00 a	61.37 g
250 mM + NSi	24.00 defg	4.88 bcd	0.83 cdefgh	4.05 abcde	4.90 ab	0.13 def	0.02 cde	0.10 bcd	7.00 ab	59.23 j

**Table 2:** Effect of silicon and nano-silicon on growth and development of *Pisum sativum* plants (flowering stage) exposed to salinity stress. Means (of three replicates), in each column, followed by similar letter are not significantly different at the 5% probability level using the Post Hoc. Duncan test.

Treatment	Shoot Length (cm plant^−1^)	Shoot Fresh Weight (g plant^−1^)	Shoot Dry Weight (g plant^−1^)	Shoot Water Content (g plant^−1^)	Root Length (cm plant^−1^)	Root Fresh Weight (g plant^−1^)	Root Dry Weight (g plant^−1^)	Root Water Content (g plant^−1^)	No of Leaves/Plant	Leaf Area (cm^2^ plant^−1^)	No of Flowers/Plant
Control	32.60 ab	5.50 ef	0.93 efg	4.57 fg	4.75 ab	0.22 a	0.04 e	0.18 a	10.00 ab	109.06 b	1.00 a
Si	34.00 ab	7.69 c	1.51 bc	6.18 cde	6.25 a	0.26 a	0.06 b	0.20 a	10.00 ab	88.34 f	2.00 a
NSi	34.10 ab	13.38 a	2.23 a	11.14 a	6.37 a	0.27 a	0.08 a	0.19 a	11.00 a	87.65 g	2.00 a
100 mM	31.13 abcd	6.98 cde	1.39 bc	5.59 cdef	4.90 ab	0.19 a	0.04 e	0.15 a	10.00 ab	104.01 c	1.00 a
100 mM + Si	31.85 abc	7.97 c	1.34 bc	6.63 c	3.60 b	0.22 a	0.05 cd	0.17 a	10.00 ab	62.00 n	2.00 a
100 mM + NSi	36.20 a	11.28 b	1.63 b	9.65 b	4.20 ab	0.25 a	0.05 bc	0.20 a	10.00 ab	68.38 j	2.00 a
150 mM	31.00 abcd	7.25 cd	1.19 cdef	6.06 cde	5.25 ab	0.23 a	0.04 de	0.19 a	10.00 ab	89.51 d	1.00 a
150 mM + Si	31.33 abcd	7.66 c	1.27 cd	6.40 cd	4.40 ab	0.25 a	0.05 cde	0.20 a	10.00 ab	68.14 k	2.00 a
150 mM + NSi	33.00 ab	10.73 b	1.63 b	9.10 b	4.50 ab	0.20 a	0.05 cd	0.15 a	10.00 ab	89.13 e	2.00 a
200 mM	25.40 def	4.98 f	0.87 fg	4.10 g	5.30 ab	0.15 a	0.04 e	0.11 a	9.00 b	76.71 h	1.00 a
200 mM + Si	25.60 def	4.65 f	0.89 fg	3.75 g	4.90 ab	0.14 a	0.04 de	0.10 a	9.00 b	67.06 l	2.00 a
200 mM + NSi	28.87 bcde	8.04 c	1.25 cde	6.80 c	5.35 ab	0.17 a	0.05 cde	0.12 a	10.00 ab	135.04 a	2.00 a
250 mM	21.40 f	4.70 f	0.85 g	3.85 g	6.20 a	0.14 a	0.04 e	0.11 a	9.00 b	66.33 m	2.00 a
250 mM + Si	23.00 ef	5.89 def	0.85 g	5.05 defg	5.30 ab	0.37 a	0.04 e	0.33 a	10.00 ab	72.85 i	2.00 a
250 mM + NSi	26.25 cdef	5.82 def	0.99 defg	4.83 efg	5.55 ab	0.13 a	0.04 e	0.09 a	10.00 ab	56.54 o	2.00 a

**Table 3:** Effect of silicon and nano-silicon on growth and development of *Pisum sativum* plants (yield stage) exposed to salinity stress. Means (of three replicates), in each column, followed by similar letter are not significantly different at the 5% probability level-using Post Hoc Duncan test.

Treatment	Shoot Length (cm plant^−1^)	Plant Height (cm plant^−1^)	Pod Length (cm plant^−1^)	Pod Weight (g plant^−1^)	Crop Yield (g plant^−1^)	No of Pods /Plant	No of Seeds /Pod	Seed Weight (g pod^−1^)	Total Seed Yield (g plant^−1^)	Straw Yield (g plant^−1^)	Harvest Index	Crop Index	Mobilization Index
Control	36.00 abc	42.35 ab	10.20 ab	6.13 c	7.94 abcd	3.00 bc	7.00 ab	3.53 abc	3.37 abcd	6.81 abc	0.48 ab	0.32 ab	0.05 a
Si	34.00 abcd	37.90 abcd	10.10 abc	8.09 b	9.02 abc	3.00 bc	7.00 ab	3.97 a	4.10 a	6.98 abc	0.59 a	0.37 a	0.05 a
NSi	39.55 a	45.10 a	10.35 a	9.91 a	11.47 a	5.00 a	8.00 a	3.92 ab	3.95 ab	8.62 a	0.46 abc	0.31 ab	0.04 a
100 mM	33.60 abcd	37.70 abcd	9.03 bcd	4.35 de	7.89 abcd	3.00 bc	6.00 ab	2.02 def	2.54 abcde	6.33 abcd	0.40 abc	0.29 abc	0.05 a
100 mM + Si	34.83 abc	38.17 abcd	10.10 abc	7.82 b	8.44 abcd	3.00 bc	7.00 ab	2.75 bcd	3.59 abc	6.93 abc	0.46 abc	0.30 ab	0.04 a
100 mM + NSi	37.00 ab	41.75 ab	9.80 abc	7.97 b	10.17 ab	4.00 ab	7.00 ab	3.74 ab	2.95 abcde	8.18 a	0.37 abc	0.27 abc	0.03 a
150 mM	32.95 abcd	36.70 bcd	8.50 de	3.46 e	5.70 cde	1.00 d	6.00 ab	1.51 defg	1.55 def	4.91 cde	0.31 abc	0.23 bc	0.05 a
150 mM + Si	34.13 abcd	37.97 abcd	9.85 abc	6.14 c	6.55 bcde	3.00 bc	7.00 ab	2.39 cde	2.42 abcde	6.14 abcd	0.47 abc	0.30 ab	0.06 a
150 mM + NSi	35.00 abc	41.4 abc	9.05 bcd	5.65 cd	7.18 bcd	3.00 bc	7.00 ab	2.48 cde	2.38 abcde	8.10 ab	0.29 bc	0.23 bc	0.03 a
200 mM	29.50 cd	35.65 bcd	8.30 de	3.42 e	4.88 de	1.00 d	6.00 ab	1.02 fg	1.44 ef	4.02 de	0.36 abc	0.26 abc	0.06 a
200 mM + Si	33.25 abcd	37.00 bcd	9.05 bcd	4.84 cde	5.64 cde	3.00 bc	7.00 ab	2.05 def	2.05 cdef	5.59 bcd	0.37 abc	0.27 abc	0.05 a
200 mM + NSi	32.10 abcd	35.80 bcd	8.90 cde	4.74 cde	5.72 cde	3.00 bc	6.00 ab	2.04 def	2.18 bcdef	6.06 abcd	0.45 abc	0.29 abc	0.07 a
250 mM	26.93 d	33.50 d	7.70 e	1.91 f	2.82 e	1.00 d	5.00 b	0.49 g	0.49 f	2.68 e	0.20 c	0.16 c	0.07 a
250 mM + Si	29.00 cd	36.50 bcd	9.00 bcd	3.88 e	4.99 de	2.00 cd	6.00 ab	1.61 defg	1.83 cdef	3.93 de	0.47 abc	0.32 ab	0.08 a
250 mM + NSi	29.67 bcd	34.00 cd	8.47 de	3.51 e	5.35 cde	3.00 bc	6.00 ab	1.31 efg	1.73 def	4.43 cde	0.39 abc	0.28 abc	0.06 a

**Table 4:** Effect of silicon and nano-silicon on Na^+^ and K^+^ contents (mg/g dry weight) and Na ^+^ /K^+^ ratio of *Pisum sativum* plants (vegetative stage) exposed to salinity stress. Means (of three replicates), in each column, followed by similar letter are not significantly different at the 5% probability level-using Post Hoc Duncan test.

Treatment	Na^+^		K^+^		Na^+^/K^+^ Ratio	
	Root	Shoot	Root	Shoot	Root	Shoot
Control	9.80 k	7.97 l	12.96 f	21.84 c	0.76 j	0.36 l
Si	10.20 j	8.90 k	11.06 h	24.89 a	0.92 h	0.36 l
NSi	9.80 k	7.97 l	16.16 d	19.78 e	0.61 m	0.40 k
100 mM	12.19 h	17.21 f	6.80 l	10.50 k	1.79 d	1.64 d
100 mM + Si	10.20 j	13.20 i	10.50 i	12.90 j	0.97 g	1.02 g
100 mM + NSi	9.80 k	11.20 j	13.20 e	15.87 h	0.74 jk	0.71 j
150 mM	20.24 c	18.21 e	9.80 j	8.32 l	2.07 c	2.19 c
150 mM + Si	12.19 h	15.22 g	10.50 i	13.22 i	1.16 e	1.15 f
150 mM + NSi	11.19 i	14.23 h	16.20 c	19.40 f	0.69 l	0.73 j
200 mM	26.23 b	19.21 d	11.80 g	3.80 m	2.22 b	5.06 b
200 mM + Si	13.24 f	23.23 b	16.20 c	19.33 g	0.82 i	1.20 e
200 mM + NSi	12.80 g	19.22 d	17.88 b	23.41 b	0.72 kl	0.82 h
250 mM	32.24 a	21.22 c	9.06 k	2.90 n	3.56 a	7.32 a
250 mM + Si	19.22 d	26.23 a	17.88 b	21.54 d	1.07 f	1.22 e
250 mM + NSi	16.20 e	19.22 d	22.25 a	24.90 a	0.73 jk	0.77 i

**Table 5:** Sequences of primers used in qPCR.

Target Gene	Forward Primer 5′–3′	Reverse Primer 5′–3′
SOD	GCGACATTCTTCCGGCTTTC	GCCCAGCCTGAACCAAATTG
POD	CTAGTTGCTCTTTCCGGTGC	GGTTTGTTCCACTTCCACCC
CAT	TCACAGGGATGAGGAGGTCA	TGGATCGGTGTCGGATAAAGC
PsLecRLK	TGAAGGATGGGAAGTGGAAG	CTCGACCGGTTTTGATCACT
Actin	GCTGTCCTCTCCCTCTATGCA	GCCGAGGTGGTGAACATATACC
Tubulin	GTACACTGGTGAAGGCATGGA	ACTGCTGAACACACTTACACG

## Data Availability

The data presented in this study are available in this article.
